# Long-term evaluation of the antimicrobial susceptibility and microbial profile of subgingival biofilms in individuals with aggressive periodontitis

**DOI:** 10.1590/S1517-838246220131037

**Published:** 2015-06-01

**Authors:** Talita Gomes Baêta Lourenço, Débora Heller, Renata Martins do Souto, Mayra Xavier e Silva-Senem, Victor Macedo Varela, Maria Cynesia Barros Torres, Eduardo Jorge Feres-Filho, Ana Paula Vieira Colombo

**Affiliations:** 1 Universidade Federal do Rio de Janeiro, Instituto de Microbiologia, Universidade Federal do Rio de Janeiro, Rio de Janeiro, RJ, Brasil, Instituto de Microbiologia, Universidade Federal do Rio de Janeiro, Brazil.; 2 Universidade Federal do Rio de Janeiro, Departamento de Clínica Dentária, Universidade Federal do Rio de Janeiro, Rio de Janeiro, RJ, Brasil, Departamento de Clínica Dentária, Universidade Federal do Rio de Janeiro, Brazil.; 3 Boston University, Oral Biology Departament, Goldman School of Medicine, Boston, MA, USA, Oral Biology Departament, Goldman School of Medicine, Boston, MA, USA.

**Keywords:** aggressive periodontitis, biofilms, microbial sensitivity tests, DNA probes

## Abstract

This study evaluates the antimicrobial susceptibility and composition of subgingival biofilms in generalized aggressive periodontitis (GAP) patients treated using mechanical/antimicrobial therapies, including chlorhexidine (CHX), amoxicillin (AMX) and metronidazole (MET). GAP patients allocated to the placebo (C, n = 15) or test group (T, n = 16) received full-mouth disinfection with CHX, scaling and root planning, and systemic AMX (500 mg)/MET (250 mg) or placebos. Subgingival plaque samples were obtained at baseline, 3, 6, 9 and 12 months post-therapy from 3–4 periodontal pockets, and the samples were pooled and cultivated under anaerobic conditions. The minimum inhibitory concentrations (MICs) of AMX, MET and CHX were assessed using the microdilution method. Bacterial species present in the cultivated biofilm were identified by checkerboard DNA-DNA hybridization. At baseline, no differences in the MICs between groups were observed for the 3 antimicrobials. In the T group, significant increases in the MICs of CHX (p < 0.05) and AMX (p < 0.01) were detected during the first 3 months; however, the MIC of MET decreased at 12 months (p < 0.05). For several species, the MICs significantly changed over time in both groups, *i.e.*, *Streptococci* MICs tended to increase, while for several periodontal pathogens, the MICs diminished. A transitory increase in the MIC of the subgingival biofilm to AMX and CHX was observed in GAP patients treated using enhanced mechanical therapy with topical CHX and systemic AMX/MET. Both protocols presented limited effects on the cultivable subgingival microbiota.

## Introduction

Generalized aggressive periodontitis (GAP) is a severe form of periodontal disease characterized by the widespread destruction of the periodontium at a high progression rate in young subjects (Armitage, 1999). The adjunctive use of antimicrobials combined with the mechanical removal of the subgingival biofilm has been demonstrated as an effective therapeutic strategy for treating GAP (Herrera *et al.*, 2002, 2008; Haffajee *et al.*, 2003). Specifically, the administration of amoxicillin (AMX) and metronidazole (MET), combined or not with the topical use of chlorhexidine (CHX), provides significant clinical and microbiological benefits for GAP patients post-therapy (Guerrero *et al.*, 2005). However, some patients with severe periodontal destruction do not respond favorably to mechanical/antimicrobial therapy (Colombo *et al.*, 1998, 2009). Treatment failure might have several causes, including the existence of subgingival microbiota resistant to the drugs of choice (Listgarten *et al.*, 1993, Mejia *et al.*, 1995). Antimicrobial resistance has become a serious problem for the treatment of a large number of infections worldwide. The inappropriate and irrational use of antimicrobials leads to the emergence, spread and persistence of resistant microorganisms, resulting in prolonged illness and greater risk of death (Gootz, 2010). Thus, an effective antimicrobial protocol for treating periodontitis should consider the severity of the disease, the general health of the host, the target microorganisms, and the pharmacokinetics, adverse effects and costs of the drug (Seymour and Hogg, 2008, Heasman *et al.*, 2011). Moreover, periodontal diseases are polymicrobial, and biofilm-related infections widely vary in microbial composition and diversity among sites and individuals with similar clinical manifestations (Socransky and Haffajee, 2002). Bacterial species growing in biofilms are less susceptible to antimicrobial action (Costerton *et al.*, 1999). Nevertheless, few studies have directly examined the *in vitro* antimicrobial susceptibility of subgingival plaques in biofilms or mixed cultures (Wright *et al.*, 1997, Eick *et al.*, 2004, Sedlacek and Walker, 2007). This assessment could provide additional information on the susceptibility of periodontal microbiota in GAP prior to the use of antimicrobials. Furthermore, subsequent evaluation of the drug administration might reveal potential changes in the resistance profile of this microbiota. Thus, the aims of the current study were to determine the bacterial composition and antimicrobial susceptibility profile of the subgingival biofilm in GAP patients before and up to 12 months after treatment with CHX, AMX, MET or placebo.

## Material and Methods

### Subject population

This study was conducted as a randomized, double-blinded, placebo-controlled, single-center, 12-month clinical trial as previously described (Heller *et al.*, 2011, Varela *et al.*, 2011, Silva-Senem *et al.*, 2013). The study protocol was approved through the Ethics in Human Research Committee of the Institute for Community Health Studies at the Federal University of Rio de Janeiro, Brazil (EHRC/ICHS-FURJ, protocol #45/2007). The subjects were selected between March 2008 and June 2009 from a pool of first-time patients referred to the Division of Graduate Periodontics of the School of Dentistry at the Federal University of Rio de Janeiro (UFRJ), Brazil. Included patients were diagnosed with GAP according to criteria of the American Academy of Periodontology (Armitage, 1999). In addition, the patients were between 18–39 years of age and had at least 16 teeth and 4 sites on different teeth (3 sites other than central incisors or first molars), with a probing pocket depth (PPD) ≥ 6 mm and clinical attachment level (CAL) ≥ 5 mm and bleeding on probing (BOP). The exclusion criteria were allergy to penicillin, MET or CHX; diabetes; immunodeficiency; required antibiotic coverage for periodontal procedures; long-term use of anti-inflammatory medication; periodontal treatment and/or use of antibiotics in the last 6 months; and pregnancy and nursing (Heller *et al.*, 2011, Varela *et al.*, 2011, Silva-Senem *et al.*, 2013).

### Clinical examination and treatment protocols

A trained and calibrated examiner (D. H.) performed clinical exams at baseline, 3, 6, 9 and 12 months post-therapy. The full-mouth clinical measurements included PPD, CAL, presence or absence of BOP, supragingival visible plaque and gingival marginal bleeding. An experienced periodontist (V.M.C.) administered periodontal treatment. The patients received full-mouth debridement with ultrasonics, complemented by the irrigation of all pockets with a 0.2% CHX gel within 24 h. Additionally, patients were instructed to rinse and gargle twice a day with a 0.12% CHX solution and brush the tongue with the same CHX gel for the next 45 days. The patients were subsequently assigned either to the test (T, systemic administration of AMX 500 mg + MET 250 mg) or the control group (C, placebo tablets). Antimicrobials or placebos were prescribed 3 times a day for 10 days, starting at the moment of assignment. In the following week, the patients were treated with staged quadrant manual scaling and root planning, followed by pocket irrigation with 0.2% CHX gel within 4–6 weeks. The patients returned at 3, 6, 9 and 12 months for clinical re-evaluation, microbiological sampling, oral hygiene evaluation, and supragingival plaque and calculus removal. Furthermore, sites with PPD > 4 mm and BOP were re-instrumented under local anesthesia (Heller *et al.*, 2011, Varela *et al.*, 2011, Silva-Senem *et al.*, 2013).

### Subgingival biofilm sampling

Subgingival biofilm samples were collected from 3–4 of the deepest sites (PPD) using individual sterile Gracey curettes (Hu-Friedy, Chicago, IL, USA). The material was pooled, placed into cryogenic tubes containing 1 mL of mycoplasma broth with 10% DMSO and stored at −20 °C.

### Determination of the MIC

Susceptibility testing was performed using the broth microdilution method according to the Clinical and Laboratory Standards Institute guidelines (CLSI, formerly NCCLS, 2004), with modifications. The pooled samples were anaerobically cultured in pre-reduced supplemented BHI broth (BBL) for 48 h at 37 °C. The mixed culture was centrifuged, and the bacterial suspension was subsequently adjusted to ~1.5 × 10^8^ colony forming units (cfu)/mL in saline solution (0.9%). A 10-μL aliquot of the suspension was dispensed into the wells of 96-well, round-bottom microtiter plates (TPP), containing 100 μL of two-fold serial dilutions of the AMX and MET antimicrobials (Sigma-Aldrich Co.). The antimicrobials were administered at final concentrations ranging from 128 to 0.25 μg/mL for AMX and MET. For CHX, 22 μL of the bacterial suspensions were placed into wells containing 88 μL of the antimicrobials diluted in PRAS-supplemented BHI broth to final concentrations ranging from 2% to 0.02%. Each microplate included positive (bacterial suspension without antimicrobial treatment) and negative controls (medium only), and all experiments were performed in duplicate. The microplates were incubated under anaerobic conditions for 48 h at 37 °C. One examiner obtained visual readings. The MIC was defined as the lowest antimicrobial concentration yielding no visual bacterial growth.

### Determination of the Composition of the Subgingival Biofilm through Checkerboard DNA-DNA Hybridization

The composition of the subgingival biofilm samples cultivated in the microplates without antimicrobials (positive controls) at baseline, 3, 6, 9 and 12 months after treatment was determined using the checkerboard method (Socransky *et al.*, 1994), with modifications (Heller *et al.*, 2011).

### Statistical analysis

A statistical program (SPSS, Statistical Package for the Social Sciences, version 19.0, IBM) was used for all analyses. The clinical and demographic features of the groups were compared using the Mann-Whitney and Chi-square tests. The MICs of each antimicrobial for each patient was averaged within the groups at all time points. Significant differences between groups and over time were examined using the Mann-Whitney, Friedman and Wilcoxon signed rank tests. For the checkerboard data, the levels of each species were computed for each sample and patient and averaged within each group. For graphic presentation, the levels (scores 0 to 5) of each species in a sample were converted to absolute numbers and log10 transformed. Comparisons between groups over time were evaluated using the Mann-Whitney and Friedman tests, whereas the differences between two time points were assessed using the Wilcoxon signed rank test. For the checkerboard analysis, adjustments for multiple comparisons were made according to Socransky *et al.* (1991). Briefly, an overall p of 0.05 = 1 − (1 − k)^54^ was computed, where k was the desired individual p value. Thus, a p value < 0.00095 was considered to be statistically significant at p < 0.05. The level of significance for all the other analyses was 5%.

## Results

**Figure 1:**
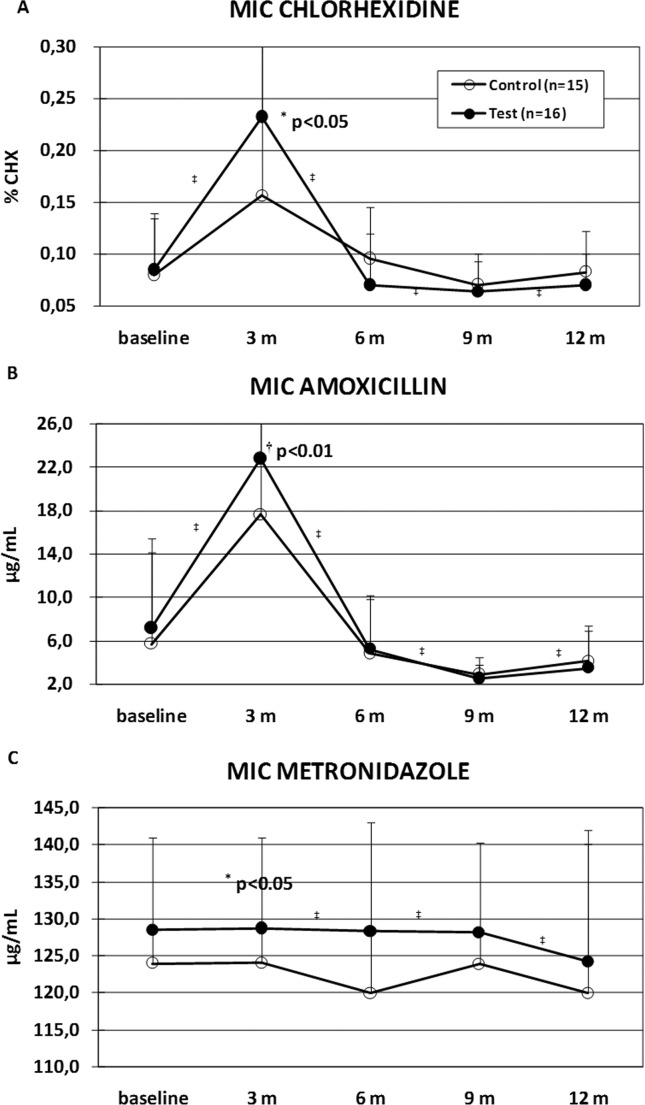
Mean (± SD) of the MICs of chlorhexidine (A), amoxicillin (B) and metronidazole (C) in the two therapeutic groups at baseline, 3, 6, 9 and 12 months after periodontal therapy. No differences between groups were observed at any time point (p > 0.05, Mann-Whitney test). Refers to significant changes over time in the test groups (Friedman test). *p < 0.05 and ^†^p < 0.01 refer to significant differences between the 3-month visit and the other time points in the test groups (Wilcoxon sign rank test).

Information on adverse events, adherence to the local and systemic antimicrobial regimen, and the demographic and full-mouth periodontal clinical features of the subjects in both therapeutic groups has been published elsewhere (Heller *et al.*, 2011, Varela *et al.*, 2011, Silva-Senem *et al.*, 2013). The MICs for the three antimicrobials in subgingival biofilm samples obtained from GAP patients before and up to 1 year after both treatment protocols are shown in Figures 1A–C. At baseline, no significant differences between groups were observed for the MICs of all tested antimicrobials (p > 0.05, Mann-Whitney test). However, significant increases in the MICs of CHX (p < 0.05, Figure 1A) and AMX (p < 0.01, Figure 1B) were detected in the T group at 3 months compared with all other time points (Friedman and Wilcoxon tests). Significant differences over time were also observed for the MIC of MET (p < 0.05, Friedman test), which decreased at 12 months post-therapy in the T group (Figure 1C). In the C group, no significant changes in the MICs of any antimicrobial were observed over time post-therapy (p > 0.05, Friedman test). Moreover, no significant differences in the MICs of CHX, AMX or MET between groups were detected at 3, 6, 9 and 12 months post-therapy (p > 0.05, Mann-Whitney test).

**Figure 2:**
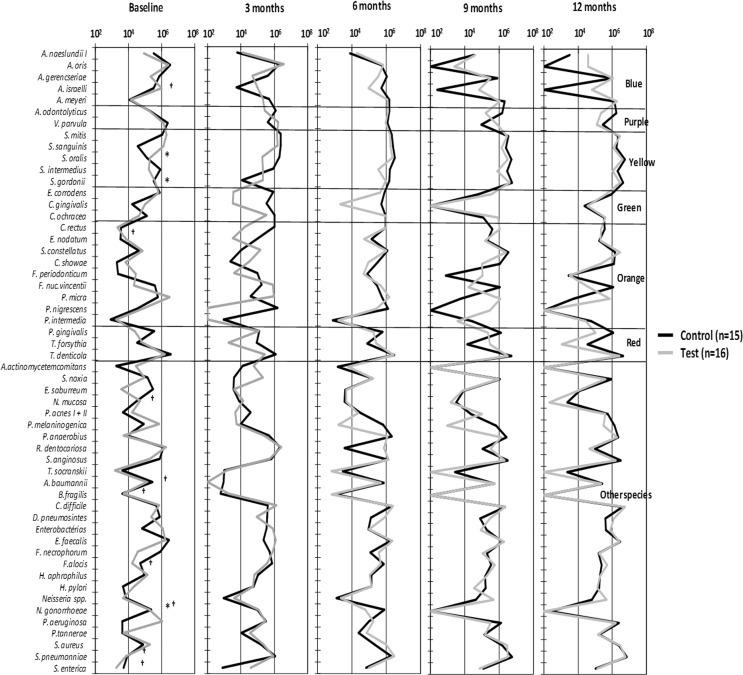
Mean levels of bacterial species in the subgingival biofilm cultivated *in vitro* from GAP subjects treated using mechanical therapy associated with CHX and AMX combined with MET (test group) or placebo (control group) at baseline, 3, 6, 9 and 12 months post-therapy. No significant differences between groups were observed for any species at any time point, after adjusting for multiple comparisons (Mann-Whitney test, p > 0.00095). *Refers to significant changes in bacterial levels over time in the control group, and ^†^ refers to significant changes in bacterial levels over time in the test groups (Friedman test, p < 0.00095). The species were ordered into different microbial complexes according to Socransky *et al.* (1998).

The composition of the subgingival biofilm cultivated *in vitro* from patients of the two clinical groups is presented in Figure 2. The species were ordered into different microbial complexes according to Socransky *et al.* (1998). The mean levels of the tested species (Table 1) were computed for both groups at each time point. At baseline, high mean levels of bacteria (4.4 × 10^5^ cells) were detected in both groups, including several periodontal pathogens. No significant differences between groups regarding bacterial mean levels were observed for any species at any time point (adjusted p < 0.00095, Mann-Whitney test). When mean counts of these species were evaluated within each group over time, few significant changes were observed (Figure 2). The numbers of *Streptococcus* spp. increased, while the number of periodontal pathogens, such as *Agreggatibacter actinomycetemcomitans*, *Tannerella forsythia*, *Parvimonas micra* and *Treponema socranskii*, diminished in both groups. However, only *Streptococcus gordonii* and *Streptococcus oralis* increased, whereas *Neisseria gonorrhoeae* significantly decreased at 12 months after treatment in the control group (Friedman test, p < 0.00095). In the test group, *Actinomyces israelli*, *Bacteroides fragilis*, *N. gonorrhoeae* and *Neisseria mucosa* were reduced, and *Acinetobacter baumannii*, *Campylobacter rectus*, *Filifactor alocis*, *Salmonella enterica* and *Streptococcus pneumoniae* significantly increased over time post-therapy (Friedman test, p < 0.00095).

**Table 1:** Bacterial taxa used for development of whole genomic DNA probes tested against subgingival biofilm samples.

Species	Strain*
*Aggregatibacter actinomycetemcomitans a*	43718
*Aggregatibacter actinomycetemcomitans b*	29523
*Aggregatibacter actinomycetemcomitans c*	625^b^
*Actinomyces gerensceriae*	23860
*Actinomyces israelli*	12102
*Actinomyces odontolyticus*	17929
*Actinomyces naeslundii*	12104
*Actinomyces oris*	43146
*Actinomyces meyeri*	35568
*Acinetobacter baumannii*	19606
*Bacteroides fragilis*	25285
*Capnocytophaga gingivalis*	33624
*Capnocytophaga ochracea*	33596
*Campylobacter rectus*	33238
*Campylobacter showae*	51146
*Clostridium difficile*	98689
*Dialister pneumosintes*	GBA27^b^
*Eubacterium nodatum*	33099
*Eubacterium saburreum*	33271
*Eikenella corrodens*	23834
*Enterococcus faecalis*	10100
*Escherichia coli*	10799
*Enterobacter cloacae*	10699
*Enterobacter sakazakii*	12868
*Enterobacter aerogenes*	13048
*Enterobacter gergoviae*	33028
*Filifactor alocis*	35896
*Fusobacterium necrophorum*	25286
*Fusobacterium periodonticum*	33693
*Fusobacterium nucleatum ss. vincentii*	49256
*Haemophilus aphrophilus*	33389
*Helicobacter pylori*	43504
*Klebsiella pneumoniae*	10031
*Klebsiella oxytoca*	12833
*Neisseria polysaccharea*	43768
*Neisseria sicca*	29256
*Neisseria subflava*	49275
*Neisseria meningitidis*	13077
*Neisseria lactamica*	23970
*Neisseria gonorrhoeae*	21824
*Neisseria mucosa*	19696
*Pantoea agglomerans*	27155
*Parvimonas micra*	33270
*Prevotella melaninogenica*	25845
*Porphyromonas gingivalis*	33277
*Prevotella intermedia*	25611
*Prevotella nigrescens*	33563
*Propionibacterium acnes I*	11827
*Propionibacterium acnes II*	43541
*Peptostreptococcus anaerobius*	27337
*Prevotella tannerae*	51259
*Pseudomonas aeruginosa*	10145
*Rothia dentocariosa*	17931
*Selenomonas noxia*	33359
*Streptococcus anginosus*	33397
*Streptococcus constellatus*	27823
*Streptococcus mitis*	49456
*Streptococcus oralis*	35037
*Streptococcus sanguinis*	10556
*Streptococcus gordonii*	10558
*Streptococcus intermedius*	27335
*Salmonella enterica sorv. typhi*	6539
*Staphylococcus aureus*	33591
*Streptococcus pneumoniae*	49619
*Tannerella forsythia*	43037
*Treponema denticola*	B1†
*Treponema socranskii*	S1†
*Veillonella parvula*	10790

* ATCC (American Type Culture Collection, Rockville, MD)

† The Forsyth Institute, (Boston, MA).

## Discussion

The use of systemic antimicrobials as adjunct treatments to mechanical therapy in GAP is controversial. There are no specific antimicrobial therapy protocols for treating different forms of periodontitis, and among the currently employed protocols, none of these therapies completely eliminated the need for retreatment (Herrera *et al.*, 2002, 2008; Haffajee *et al.*, 2003). The systemic administration of antimicrobials should always consider the risk-benefits for the patients, particularly the costs and adverse effects of additional drugs (Seymour and Hogg, 2008, Heasman *et al.*, 2011). In general, the restricted use of systemic antimicrobials is the best strategy to avoid the increase in resistance worldwide (Enne, 2010). Thus, alternative approaches of intensive mechanical debridement combined with topical antimicrobials, such as CHX, have been attempted for treating severe forms of periodontitis (Quirynen *et al.*, 1995; Sigusch *et al.*, 2005). In previous studies (Heller *et al.*, 2011, Varela *et al.*, 2011, Silva-Senem *et al.*, 2013), we compared the clinical and microbiological efficacy of an enhanced non-surgical mechanical therapy with the extensive use of topical CHX associated with systemic AMX and MET or placebos for up to one year. The findings indicated that both therapeutic approaches were efficient in improving clinical parameters and reducing periodontal pathogens. Moreover, we also examined how these treatments would affect the susceptibility profile and composition of cultivable subgingival microbiota over time. Conventional *in vitro* tests of antimicrobial susceptibility are typically performed in planktonic pure cultures (Wright *et al.*, 1997; Eick *et al.*, 2004, Sedlacek and Walker, 2007), which do not reflect the complex polymicrobial nature of the subgingival microbiota (Socransky and Haffajee, 2002). Developing heterotypic biofilm models for testing antimicrobial agents is a complex task, and the number of different species co-existing in an *in vitro* model is often limited. Although we did not employ a mixed biofilm model for evaluating the susceptibility profile, we directly determined the MICs for the 3 antimicrobials in mixed cultures of subgingival biofilm samples obtained from each patient pre- and post-therapy using the microdilution method for anaerobes (NCCLS, 2004). Given that there are no standardized protocols for antimicrobial testing in anaerobic mixed culture, it is important to interpret these results with caution. At the pre-therapy phase, we observed that the susceptibility of the cultivable subgingival microbiota was similar in both groups. The mean MICs of AMX and CHX were lower than the plasmatic and gingival crevicular fluid concentrations typically observed after the systemic administration of 500 mg of AMX 3 times per day (5–8 μg/mL) (Kleinfelder *et al.*, 1999) and the topical use of CHX (0.12 and 0.2%). In contrast, the MIC of MET was much greater than the concentrations detected in plasma and gingival crevicular fluid (13–14 μg/mL) after a dosage of 500 mg administered 3 times per day (Pähkla *et al.*, 2005). Other authors have also reported high MICs for MET in strains of *Prevotella* spp., *P. gingivalis*, *Fusobacterium* spp. and *A. actinomycetemcomitans* isolated from chronic periodontitis patients in Colombia (Serrano *et al.*, 2009, Ardila *et al.*, 2010). Moreover, when Spanish and Dutch patients were compared, higher proportions of *F. nucleatum* and *A. actinomycetemcomitans* isolates resistant to MET and other commonly used antimicrobials were observed (Van Winkelhoff *et al.*, 2000). The abusive use of antimicrobials and poor patient compliance, particularly in developing countries (Berquo*et al*., 2004), may be responsible for the variability in the susceptibility of the periodontal microbiota in subjects from distinct populations, suggesting that a single antimicrobial protocol to treat periodontitis might not be adequate for all patients (Teles *et al.*, 2006). However, the narrow spectrum of MET for strict anaerobes (Seymour and Hogg, 2008) might limit the *in vitro* effect of this drug on the mixed culture of subgingival plaques.

After systemic treatment with AMX and MET, a significant but transitory increase in the MICs of AMX and CHX was observed in the T group. Although a similar pattern was detected in the placebo group, the changes in the MICs of all antimicrobials over time were not significant for this group. Interestingly, topical CHX was extensively used in both groups, but the increase in the MIC of this antimicrobial was significant only in the T group. Conceivably, the systemic administration of AMX and/or MET might have a synergistic impact on the susceptibility of the microbiota to CHX, reflecting ecological shifts in the periodontal microbiota. Other studies have also reported the selective and transient pressure of systemic antimicrobials on the susceptibility of the subgingival microbiota (Feres *et al.*, 2001, Buchmann *et al.*, 2003, Rodrigues *et al.*, 2004). A decrease in the MIC of MET was observed in both groups, although the concentrations remained high at 12 months after treatment. The unusual occurrence of resistance to MET has been associated with technical problems during cultivation under anaerobic conditions (Roberts, 2002, Diniz *et al.*, 2004). Nevertheless, genes associated with MET resistance have been determined in *Bacteroides* spp. (Trinh*et al*., 1996). In addition, periodontal pathogens cultivated in biofilms are 100 times more resistant to MET compared with planktonic cultures (Wright *et al.*, 1997; Eick *et al.*, 2004; Sedlacek and Walker, 2007).

The composition of the cultivable periodontal microbiota was evaluated before and after treatment in both groups. At baseline, high levels of many of the tested bacterial species, including periodontal pathogens, were detected in the periodontitis-related biofilm in both groups, consistent with previous studies (Socransky *et al.*, 1998, Socransky and Haffajee, 2002, 2005). In general, an increase in *Streptococcus* spp. and a reduction of several pathogenic species in the T and C groups were observed. These changes are consistent with the establishment of a microbiota compatible with periodontal health following mechanical therapy with or without the use of systemic antimicrobials (Colombo *et al.*, 2005, Teles *et al.*, 2006). Regarding non-oral bacterial pathogens, the T group presented a significant increase in the levels of several of these species (*A. baumannii*, *F. alocis*, *S. enterica* and *S. pneumoniae*) over time. Many of these microorganisms have been associated with nosocomial infections, biofilm infections and multi-resistance to antimicrobial agents. The therapeutic protocols used in the present study might be more effective against oral pathogens, but these methods might also have a limited effect on other non-oral pathogenic bacteria. The role of these species in the etiology and pathogenesis of periodontitis is unclear, although these bacteria have been frequently detected in the subgingival biofilms of subjects with periodontal diseases (Colombo *et al.*, 1998, 2002, 2009; Fritschi*et al*., 2008, Heller *et al.*, 2011, Silva-Senem *et al.*, 2013). The presence of these pathogens in subgingival biofilms might also have medical implications, as pathogenic species colonizing the periodontal biofilm might be more resistant to antimicrobials. Previous studies have suggested that major clinical and microbiological changes after mechanical therapy with or without antimicrobials are typically more pronounced in the first 3 months after therapy (Xajigeorgiou *et al.*, 2006, Mestnik *et al.*, 2010, Yek *et al.*, 2010). However, as shown in figure 2, a few species continued to diminish after 9 and 12 months in both groups. For example, *A. actinomycetemcomitans* and *P. nigrescens* were not detected in the cultivated biofilm at 9 and 12 months after both treatment protocols. The reinforcement in oral hygiene and re-instrumentation during the monitoring visits might have contributed to the continuous reduction of certain pathogenic species.

Thus, these data indicate that enhanced mechanical periodontal therapy associated with the extensive topical use of CHX and systemic administration of AMX and MET leads to a transitory increase in the MICs of the subgingival biofilm to AMX and CHX. Notably, resistance was not evaluated in the present study because there are no breakpoints to assess susceptibility or resistance when MICs are obtained upon biofilm analysis. Both therapeutic protocols presented similar and limited effects on the composition of the cultivable subgingival microbiota over time. Given the similar clinical benefits of both approaches (Heller *et al.*, 2011, Varela *et al.*, 2011, Silva-Senem *et al.*, 2013), the enhanced mechanical periodontal therapy associated with the topical use of CHX may be suggested as a potential and effective alternative for the treatment of individuals with GAP, without major implications on the susceptibility profile of the periodontal microbiota.
